# Probiotic Supplements: Hope or Hype?

**DOI:** 10.3389/fmicb.2020.00160

**Published:** 2020-02-28

**Authors:** Yuxuan Wang, Yinyin Jiang, Yuxin Deng, Chen Yi, Yangcan Wang, Mengnan Ding, Jie Liu, Xuanjing Jin, Lishan Shen, Yue He, Xinyun Wu, Xuefei Chen, Changyi Sun, Min Zheng, Ruijia Zhang, Hailv Ye, Huiting An, Aloysius Wong

**Affiliations:** Department of Biology, College of Science and Technology, Wenzhou-Kean University, Wenzhou, China

**Keywords:** probiotics, antibiotic resistance, health/dietary supplements, horizontal gene transfer, adaptive evolution, *Lactobacillus*, lactic acid bacteria

## Abstract

Probiotic bacteria have been associated with various health benefits and included in overwhelming number of foods. Today, probiotic supplements are consumed with increasing regularity and record a rapidly growing economic value. With billions of heterogeneous populations of probiotics per serving, probiotic supplements contain the largest quantity of probiotics across all functional foods. They often carry antibiotic-resistant determinants that can be transferred to and accumulate in resident bacteria of the gastrointestinal tract and risk their acquisitions by opportunistic pathogens. While the health benefits of probiotics have been widely publicized, this health risk, however, is underrepresented in both scientific studies and public awareness. On the other hand, the human gut presents conditions that are unfavorable for bacteria, including probiotics. It remains uncertain if probiotics from supplements can tolerate acids and bile salts that may undermine their effectiveness in conferring health benefits. Here, we put into perspective the perceived health benefits and the long-term safety of consuming probiotic supplements, specifically bringing intolerance to acids and bile salts, and the long-standing issue of antibiotic-resistant gene transfer into sharp focus. We report that probiotics from supplements examined in this study have poor tolerance to acids and bile salts while also displaying resistance to multiple antibiotics. They could also adapt and gain resistance to streptomycin *in vitro*. In an environment where consuming supplements is considered a norm, our results and that of others will put in perspective the persisting concerns surrounding probiotic supplements so that the current hype does not overpower the hope.

## Introduction

**FIGURE 1:**
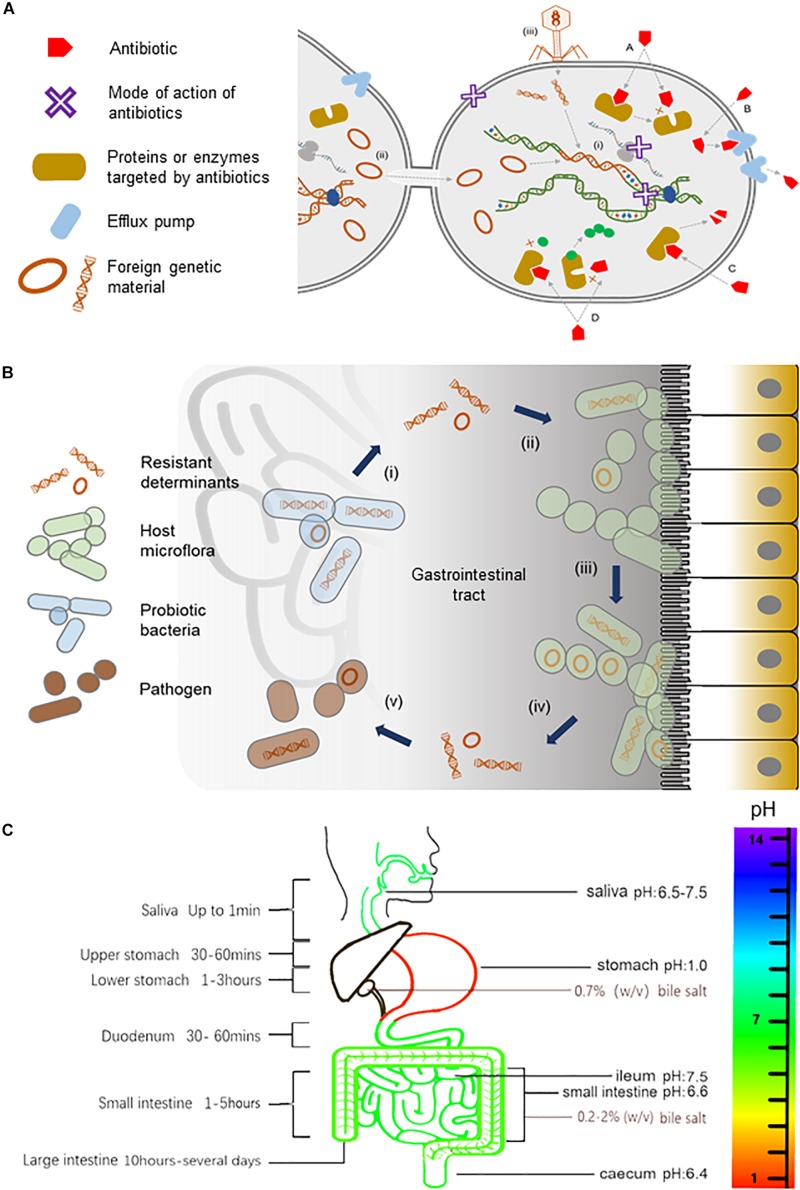
Antibiotics and their resistance in the human gut. **(A)** An illustration of the mode of actions of antibiotics, the transmission of resistant determinants, and the biochemistry of antibiotic resistance. Antibiotics, either bacteriostatic or bactericidal, can exert their effects through the inhibition of enzymes involved in cell wall synthesis, the inhibition of protein translation at small or large ribosomal subunits, or the inhibition of nucleic acid metabolism in the bacterial cell as represented by a purple X in the diagram. Bacteria gain resistance to antibiotics through (i) mutations to the bacteria genome or by acquiring mobile elements via horizontal gene transfer including (ii) conjugation events involving sexual pilus or (iii) direct uptake of DNA fragments by transformation or with the help of bacteriophages in (transduction events. The biochemistry of resistance includes (A) altering antibiotic target site, (B) reduced antibiotic uptake, (C) inactivation of antibiotic, and (D) producing alternative targets that are resistant to antibiotics. **(B)** A model for the trafficking of resistant genes in the human gut. Probiotic bacteria from supplements that harbor resistant determinants (i) can transfer their mobile elements to host gut bacteria (ii). Gut microflora, over time, can build up a reservoir of antibiotic-resistant genes (iii), which can, in turn, be transferred (iv) to opportunistic pathogens (v) within the gastrointestinal tract, thus rendering antibiotics ineffective (illustration adapted from Zheng et al., 2017). **(C)** The pH, bile salts, and transit time of food in the gastrointestinal tract. The pH in saliva is between 6.5 and 7.5. The pH can reach as low as 1.0 in the stomach, rising to 6.6 in the proximal small intestine and 7.5 in the ileum before falling sharply to 6.4 in the cecum. Bile salts in the gallbladder are approximately 0.7% (wt/vol), but in the small intestine, it ranges between 0.2 and 2% (wt/vol) (Kristoffersen et al., 2007). During transit, food is exposed to saliva for up to 1 min and gastric juice for 1.5 to 4 h. Food transits through the small intestine for approximately 1 to 5 h and remains in the large intestine for 10 h to several days.)

In the natural environments, antibiotics are found in much lower concentrations than that used in therapeutics. They are thought to serve as signals that enable the host to perform various physiological functions including intercellular communication, regulation of cellular metabolism, triggering intracellular transcriptional changes in response to stresses, and inhibiting the growth of competing bacteria populations (Haas and Défago, 2005; Yim et al., 2006, 2007; Fajardo and Martínez, 2008; Romero et al., 2011). Antibiotics act by inhibiting enzymes directly involved in cell wall and protein or nucleic acid metabolisms [see reviews by O’Connell (2001), Martinez et al. (2009), Kohanski et al. (2010), Du Toit (2018)]. Correspondingly, bacteria have intrinsic cellular mechanisms that shield them against the very same antibiotics that they produce [see reviews by Hawkey (1998), O’Connell (2001), Martinez et al. (2009), Stokes and Gillings (2011), Blair et al. (2014), Lin et al. (2015)] (Figure 1A). Genes that encode for resistance can be acquired through mobile elements such as plasmids and transposons, transformation or transduction via bacteriophages, or spontaneous mutations that repurpose existing efflux pumps or enzymes to recognize antibiotics (Woodford and Ellington, 2007; Bennett, 2008; Stokes and Gillings, 2011; Bielecki et al., 2012; Gutierrez et al., 2013; Balcazar, 2014; Colavecchio et al., 2017; Jiang et al., 2017; Figure 1A).

Consumption of probiotics in large amounts and high frequency in the form of health or dietary supplements can accelerate these natural ways of acquiring resistance. Probiotics are associated with increasing health benefits ranging from improving nutrient absorption and immune system (Perdigon et al., 2001; Kaur et al., 2002) to preventing cancer (Hirayama and Rafter, 2000) and cardiovascular-related diseases (Nagpal et al., 2012; Kechagia et al., 2013). As such, probiotics have been included in overwhelming number of foods such as yogurt, cheese, milk, and ice cream (Homayouni et al., 2012). In reflection of their popularity, current market survey has placed probiotic supplements as the driving force of the functional foods industry that is projected to reach 64 billion USD by 2023 (Zheng et al., 2017). Today, probiotic supplements represent the largest quantity of probiotic bacteria consumed by people across all food categories as they contain billions of often heterogeneous populations of probiotic bacteria per serving. Furthermore, commercial probiotic strains are deliberately engineered to carry resistant determinants during the manufacturing process for selection purpose. The resistant genes carried on mobile elements of probiotics can be transferred by horizontal gene transfer to resident gut bacteria (Broaders et al., 2013), which over time can accumulate resistant determinants that may in turn be transferred to opportunistic pathogens within the gastrointestinal tract (Figure 1B). This health risk has also been raised by others (Gueimonde et al., 2013; Penders et al., 2013; Rolain, 2013; Hu et al., 2014; Sharma et al., 2014; van Schaik, 2015; Wong et al., 2015; Imperial and Ibana, 2016; Duranti et al., 2017; Zheng et al., 2017; Lerner et al., 2019). Based on studies conducted in other probiotic foods, it is likely that probiotic supplements pose a similar if not an even higher risk of trafficking resistant determinants because of the significantly higher amounts of probiotics consumed. Unlike other categories of functional foods, very little is known about the long-term safety of consuming such high amounts of probiotic bacteria, as studies on this category of functional food are underrepresented.

On the other hand, probiotic bacteria must navigate an arduous journey beginning in the mouth to the stomach and throughout the gastrointestinal tract in order to effectively confer health benefits (Figure 1C). For instance, the pH can reach as low as 1.0 in the stomach, rising to 6.6 in the proximal small intestine and 7.5 in the ileum before falling sharply to 6.4 in the cecum, which is inhibitory to most bacteria, including probiotics (Evans et al., 1988). Bile salts have also been shown to inhibit the growth of probiotics (Liong and Shah, 2005; Succi et al., 2005; Kristoffersen et al., 2007; Sahadeva et al., 2011; Hassanzadazar et al., 2012). Unlike the health claims of probiotic supplements, their tolerance to acids and bile salts remains uncertain, and in cases where these properties are examined, the results were inconsistent, contradictory, strain dependent, transient, and limited to *in vitro* conditions (Liong and Shah, 2005; Kristoffersen et al., 2007; Sahadeva et al., 2011). Furthermore, probiotic strains are often studied in pure homogeneous cultures where absence of other probiotics and/or resident bacteria in the gut can skew the results to favor the interest of manufacturers. Additionally, a recent review has highlighted other concerns associated with probiotics such as inadequate or problematic research design, incomplete reporting, lack of transparency and regulations of probiotics, and underreported safety of probiotics consumption (Lerner et al., 2019). Therefore, probiotic supplements may not only be ineffective, they may also be counterproductive and even detrimental, considering the clinical risk and escalating reports of antibiotic resistance globally. Here, we put into perspective the perceived health benefits and the long-term safety of consuming probiotic supplements by bringing intolerance to acids and bile salts and the long-standing issue of antibiotic resistance into sharp focus.

## Probiotics From Health Supplements Have Poor Tolerance to Acids and Bile Salts

**FIGURE 2:**
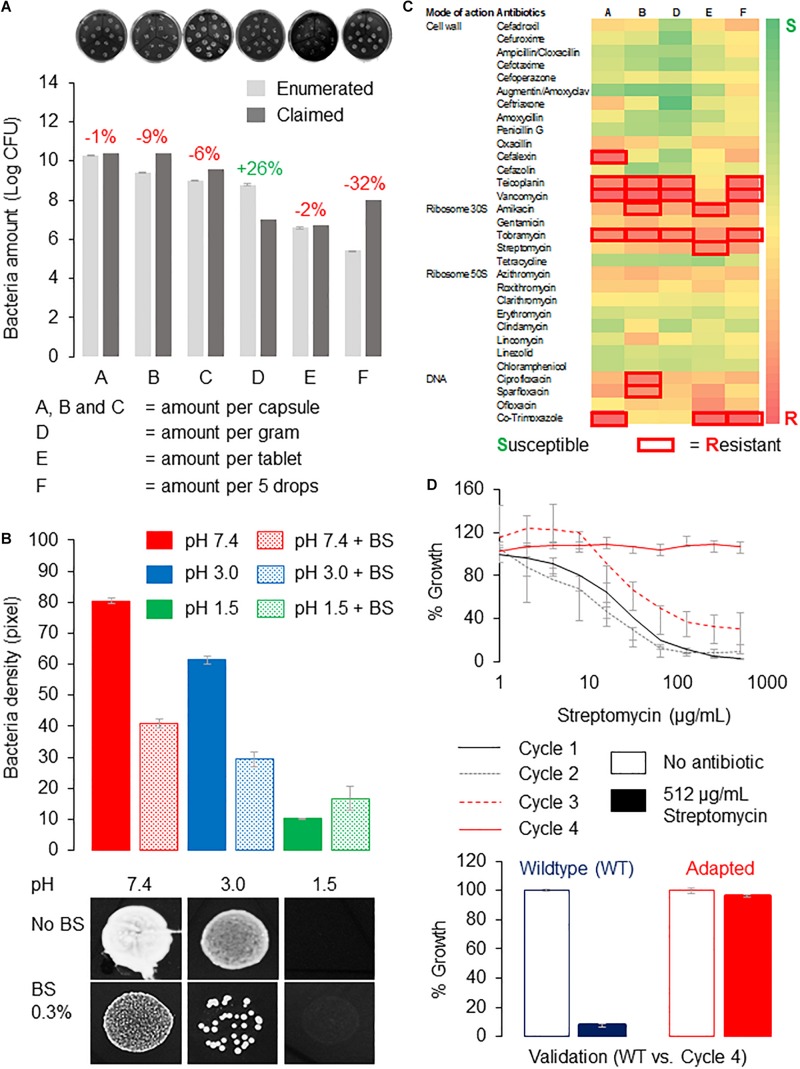
Tolerance of probiotics from supplements to gut conditions and antibiotics. **(A)** Bacteria enumeration reveals amounts comparable to that claimed by the manufacturers except for product F. Enumerated amounts were expressed as percentage of that claimed by manufacturers, where red indicates fewer and green indicates higher than claimed. Probiotic bacteria cultured on MRS agar using the drop plate method were incubated overnight at 37°C, and the number of bacteria colonies was estimated using ImageJ (Schneider et al., 2012) from high-resolution images. Representative images of bacteria recovered from the probiotic supplements are shown in the top panel. **(B)** Probiotics from product B showed poor tolerance to acidic pH and bile salts. Probiotics were dissolved in PBS adjusted to the respective pHs with or without 0.3% (wt/vol) bile salts before dropping onto MRS agar plate and incubated at similar conditions. Bacteria amounts were (estimated by measuring the density of bacteria (by pixel) using ImageJ (Schneider et al., 2012) from high-resolution photos. Bacteria growth at pH 7.4 with no bile salts was used as control. Growth of probiotic bacteria from product B dropped 25 and 87.5% at pH 3.0 and 1.5, respectively, whereas the presence of bile salts reduced bacteria viability by approximately 50% at pH 7.4 and 3.0 respectively. Representative images of bacteria spots from product B in the respective treatments are shown in the bottom panel. **(C)** A heat map of antibiotic-resistant profiles of probiotics. Probiotics dissolved in PBS were spread onto MRS agar, and antibiotic susceptibility tests were performed using commercially available antibiotic discs (HiMedia; India). Inhibition zones with diameter larger than 12 mm (i.e., twice the diameter of the disc) were considered “resistant” (boxed in red). All experiments were performed in triplicates, and error bars indicate standard error of the mean. **(D)** Probiotic bacteria from product B can adapt to high dosage of streptomycin *in vitro*. Probiotics were cultured in MRS broth containing a gradient of streptomycin (0–512 μg/mL), and bacteria growth was estimated by reading the absorbance at 600 nm (cycle 1). Wells containing bacteria of at least 80% of the no-antibiotic control were selected for the subsequent cycle(s) of adaptive evolution until 512 μg/mL of streptomycin or the highest possible concentration of streptomycin where 80% growth can be achieved (Jahn et al., 2017). Probiotics from product B were able to achieve near 100% growth at 512 μg/mL of streptomycin after four cycles of adaptive evolution.)

Here, we tested several representative probiotic supplements for their tolerance to acids and bile salts. The probiotic supplements contained predominantly *Lactobacillus* strains and were manufactured in China or Canada. The supplements were designated as A, B, C, D, E, and F, and their probiotic strains are listed in Supplementary Table 1. First, *Lactobacillus* strains from the capsules or tablets were recovered on de Man, Rogosa, and Sharpe (MRS) agar to determine if the probiotics are viable. We then enumerated the viable bacteria using the drop-plate method and compare that to the amounts on the labels. We recovered more than 90% of bacteria for products A, B, C, and E at 99, 91, 94, and 98%, respectively, whereas product D contained 128% enumerated amounts of bacteria. Thus, all products contained live bacteria with amounts comparable to that claimed by the manufacturers except for product F, which recovered only 68% live bacteria (Figure 2A). Because product F is in liquid form, it may not preserve the probiotics, as well as others that were in solids or desiccated forms. All products except product F yielded viable bacteria counts exceeding 10^6^ colony-forming units per serving, which is the suggested probiotics amount deemed sufficient to confer health benefits (Shah, 2000).

Next, we checked if probiotics from these supplements can tolerate acids and bile salts by treating them to a pH range of 1.5 to 4.5, with pH 7.4 as the control. In this test, we dissolved the products in phosphate-buffered saline (PBS) adjusted to the respective pHs and with or without the presence of 0.3% (wt/vol) bile salts. The dissolved products were dropped onto MRS agar plates and incubated at 37°C for 24 h. We took high-resolution images and used ImageJ (Schneider et al., 2012) to measure pixel intensities of bacteria spots, which correlate with their amounts or densities. We found that probiotic bacteria from product B grew poorly in acidic conditions, and they performed even worse in the presence of bile salts. In particular, the growth of probiotic bacteria dropped 25 and 87.5% at pH 3.0 and 1.5, respectively, whereas the presence of bile salts reduced bacteria viability by approximately 50% at pH 7.4 and 3.0, respectively (Figure 2B). We observed a similar trend in all products tested (data not shown). Therefore, we concluded that probiotics from the examined supplements have poor tolerance to acids and bile salts.

As tolerance to gastrointestinal conditions is crucial for bacteria to be considered as probiotics, many new *Lactobacillus* isolates from fermented foods and animals have been tested for tolerance to acids and bile salts, and they showed varying degrees of intolerance. For instance, a previous study on *Lactobacillus* strains isolated from a traditional Iranian cheese showed that only 1 of 28 isolates tolerated acids (pH 3.0) and bile salts [0.3% (wt/vol)] (Hassanzadazar et al., 2012). Similarly, 11 *Lactobacillus* strains obtained from collection centers in Australia also showed varying intolerance to acids and bile salts (Liong and Shah, 2005). It is common for commercial probiotic strains such as *Lactobacillus acidophilus* NCFM (Wang et al., 2018) and *Lactobacillus rhamnosus* GG (Corcoran et al., 2005) to be added with sugars or proteins for different buffering capacities, and combined with modern encapsulation technologies (Gbassi and Vandamme, 2012), they can increase the survivability of probiotics transiting in the gastrointestinal tract. Yet, reports of poor tolerance to acids and bile salts of commercial probiotic strains isolated from functional foods (Liong and Shah, 2005; Succi et al., 2005; Sahadeva et al., 2011; Hassanzadazar et al., 2012; Nagyzbekkyzy et al., 2016) suggest that the improvements may be too minimal or transient to be meaningful *in vivo*. For instance, bacterial strains were considered to be tolerant under short exposures (e.g., 1–2 h) to acids and bile salts (Liong and Shah, 2005; Nagyzbekkyzy et al., 2016), whereas in the gastrointestinal tract, probiotics may be exposed to acidic conditions for approximately 5 to 8 h (Maurer et al., 2015). As such, it remains uncertain if and to what degree probiotics from supplements can confer the claimed health benefits.

## Antibiotic Resistance in Probiotics From Health Supplements

Arguably the biggest health concern of consuming probiotic supplements is the risk of transferring resistant determinants. Here, we screened the probiotic supplements for susceptibility to more than 30 types of antibiotics representing different modes of actions (Figure 2C). We employed the disc diffusion method for this study using commercially available antibiotic discs (HiMedia, India). We spread probiotics dissolved in PBS onto MRS agar plates to create an even lawn of bacteria and incubate at similar conditions. Inhibition (clear) zones forming around the antibiotic discs would indicate failure of bacteria to grow around that particular antibiotic, and those with diameters of less than 12 mm, that is, twice the diameter of the disc, are considered “resistant.” We presented our data as a heat map in Figure 2C, where green represents susceptible and red represents resistant to the antibiotic. Based on Figure 2C, product B is resistant to six antibiotics, namely, teicoplanin, vancomycin, amikacin, tobramycin, ciprofloxacin, and sparfloxacin, which have different modes of actions, for example, inhibiting cell wall, protein, or DNA synthesis. Interestingly, there were no probiotics resistant to antibiotics that act on ribosome 50S.

Previous studies have shown that antibiotic-resistant genes can be transferred from one probiotic to another and from probiotics to pathogens *in vitro* (Tannock et al., 1994). Crucially, in the gut of mice, high transfer frequencies of vancomycin-resistant gene from *Enterococcus* to *L. acidophilus* were detected (Mater et al., 2008). Also in mice, a recent report has detected the coexistence of *erm(B)* as well as *tet* genes and transposons on single plasmids of three *Lactobacillus* strains. The authors demonstrated the transfer of these resistant genes to pathogens in mice upon successful colonization of the gut (Thumu and Halami, 2019). These reports are not surprising, given that relatively stable conditions in the gut, such as temperature, nutrients, and food debris, as well as the presence of a rich microflora, favor horizontal gene transfers (Lerner et al., 2017). In the event of an infection, pathogens that have acquired resistant genes may become resistant to and eventually exhaust the options of antibiotics. Because of the high amounts of probiotics consumed through probiotic supplements and their increasing popularity, the clinical ramifications and the long-term implications to human health have become more significant (Zheng et al., 2017; Kothari et al., 2019).

In addition to evidence from mice models, native human gut bacteria have also been shown to harbor various antibiotic-resistant genes. One study that compared the resistome of *Bifidobacterium* in newborn infants and in older children or adults showed that antibiotic-resistant genes are not only prevalent in gut *Bifidobacterium*, but they also built up a more robust arsenal of antibiotic-resistant genes in older children and adults (Duranti et al., 2017). Current technologies employing metagenomics approaches have enabled the diversity and dynamics of antibiotic-resistant genes in the gut microbiota to be studied and compared across populations (van Schaik, 2015). For instance, a study of 162 individuals has identified 1,093 antibiotic-resistant genes, of which Chinese individuals harbor the highest amount of antibiotic-resistant genes, followed by Danish and Spanish individuals (Hu et al., 2014).

In 2016, Imperial and Ibana described the effects of probiotics as a “‘double-edged” sword that is skewed toward the benefits in, for example, human health and agriculture, while underestimating the risk associated with trafficking antibiotic-resistant determinants. Given the lack of regulations for probiotic use especially in human health and agriculture activities, they advocated for the screening of probiotics used in livestock and human applications to reduce the spread of antibiotic-resistant genes (Imperial and Ibana, 2016) since they have already been detected in probiotic strains from foods. For instance, genes conferring resistance to tetracycline, erythromycin, aminoglycoside, β-lactam, macrolide, and chloramphenicol have been found in *Lactobacillus*, *Bifidobacterium*, and/or *Bacillus* probiotic strains (Gueimonde et al., 2013). Commercial probiotic strains, mostly *Lactobacillus* and *Bifidobacterium*, which are resistant to antibiotics and their corresponding resistant determinants have been reported in fermented sausages and vegetables, cheese, milk culture, yogurts, and drink beverages (Sharma et al., 2014). Notably, in only one known previous screening attempt on probiotics from supplements, resistance to streptomycin, vancomycin, and ciprofloxacin was reported. No resistance to erythromycin, clindamycin, tetracycline, and ampicillin was detected, and this is consistent with the results in this study. However, resistance to cephalexin, which was not found in the previous study, was detected here (Wong et al., 2015). It is increasingly clear that antibiotic-resistant genes are ubiquitously present in probiotics across all food categories and in the human microbiota. Interestingly, a correlation between the abundance of antibiotic-resistant genes in animal and in human guts has been demonstrated (Rolain, 2013; Hu et al., 2014). These reports have shown that foods, especially those treated with antibiotics and/or in which antibiotic-resistant genes were deliberately added, can establish a reservoir of antibiotic-resistant genes in the microbiota of the human gut (Gueimonde et al., 2013; Penders et al., 2013; Rolain, 2013; Hu et al., 2014; Sharma et al., 2014; van Schaik, 2015; Wong et al., 2015; Imperial and Ibana, 2016; Duranti et al., 2017; Zheng et al., 2017; Lerner et al., 2019).

Next, we investigate if probiotic bacteria from supplements can acquire resistance to a particular antibiotic using an adaptive evolution approach detailed in Jahn et al. (2017). Because product B contained probiotics displaying resistance to most antibiotics, it was selected for this adaptive evolution study. Importantly, product B did not show resistance to streptomycin, which was the antibiotic selected for this study. Briefly, we treated the probiotic bacteria grown in MRS broth on 96-well plates, to a gradient of streptomycin (0–512 μg/mL), and read the absorbance at 600 nm, which correlates with their growth. We considered this as one progressive evolution cycle (cycle 1). Those showing growth of at least 80% of that in the no-antibiotic wells were selected for the next cycle (cycle 2) of antibiotic gradient treatment. We demonstrate that probiotics from product B have adapted to streptomycin (512 μg/mL) after only four cycles of laboratory adaptive evolution (Figure 2D). We also validated the adapted bacteria comparing their tolerance to 512 μg/mL of streptomycin to that of the wild type and observed that the adapted probiotic bacteria but not the non-adapted bacteria from product B could reach near 100% growth at 512 μg/mL of streptomycin (Figure 2D). This proof-of-concept study highlights the remarkable ability of probiotics from supplements to adapt quickly and gain resistance to antibiotics.

## Discussion

Following this study, adaptation of probiotics from supplements to other classes of antibiotics should be attempted using similar approaches. We speculate that under antibiotic pressure probiotics may reduce growth and divert resources for survivability. Therefore, probiotics may fail to “climb” the antibiotic gradient or grow much slower at higher doses even after several cycles of adaptive evolution. If this is true, it is conceivable that probiotic bacteria can still shuttle resistant determinants in the gut. Comparative genomics approach, for example, metagenomics sequencing or similar genome-level assessments advocated by others, will be able to reveal the functional gene groups and metabolic pathways of antibiotic-treated probiotics (Zheng et al., 2017; Das et al., 2020; Evanovich et al., 2019; Fontana et al., 2019). To circumvent this problem, resistant determinants from probiotic strains such as those from *Lactobacillus reuteri* (ATCC 55730) (Rosander et al., 2008) could be removed prior to commercial applications in therapeutics and foods.

Besides the risk of trafficking antibiotic-resistant genes, probiotics may even be detrimental to at-risk groups such as newborns and those with weakened immunity. In vulnerable individuals, including persons with diabetes, those with cancer, and organ transplant patients, probiotics may cause localized and/or systemic infections that could lead to organ failure upon penetrating the intestinal mucosa and into the bloodstream (Kothari et al., 2019). Localized effects of probiotics include metabolic disturbances leading to opportunistic infections caused by bacteriophages virulence factors and metabolites of probiotics, as well as establishment of probiotic mobilome, gas, and bloating of the intestine as a result of intestinal probiotic overgrowth (Zmora et al., 2018; Lerner et al., 2019). Systemic effects of probiotics may include autoimmune diseases resulting from probiotics-triggered cytokine production, D-lactic acidosis, and brain fogginess (Kothari et al., 2019; Lerner et al., 2019). In light of these adverse effects, labels on probiotic supplements should contain results of clinical studies and warnings for at-risk groups, while governing bodies such as the Food and Drug Administration should check for misconducts including overestimation of bacteria, misidentification of probiotic strains, and unsupported health claims on product labels because these are common issues in food supplements (Berner and O’Donnell, 1998; Hamilton-Miller et al., 1999).

## Conclusion

In conclusion, we have highlighted the intolerance of probiotics from supplements to acids and bile salts in addition to their resistance (and adaptation) to antibiotic(s). In an environment where consuming supplements is considered a norm, our results and that of others will put in perspective the persisting concerns surrounding probiotic supplements so that the current hype does not overpower the hope.

## Data Availability Statement

The datasets generated for this study are available on request to the corresponding author.

## Author Contributions

AW conceived this project, wrote the manuscript and made the figures, and acquired the funding. AW, XW, XC, and CS performed the bacterial enumeration. AW, MZ, RZ, HY, and HA performed the antibiotic susceptibility test. AW, YaW, YJ, YuW, MD, JL, XJ, LS, and YH performed the adaptive evolution study. AW, YuW, YJ, YD, and CY analyzed the data and made the figures. AW, HY, and HA supervised and managed the project. All authors prepared, read and approved the final version of the manuscript.

## Conflict of Interest

The authors declare that the research was conducted in the absence of any commercial or financial relationships that could be construed as a potential conflict of interest.
