# Supplementary material for: Probiotic Supplements: Hope or Hype?
Source: Front Microbiol. 2020 Feb 28;11:160. doi: 10.3389/fmicb.2020.00160 (PMC7058552; doi:10.3389/fmicb.2020.00160)
Supplement: Supplementary file 1 [file Table_1.pdf]

Supplementary Material

Supplementary Table 1. Information listed on the labels of probiotic supplements.

Product	Probiotic strain	Probiotic amount (x 10^6 CFU / capsule or tablet)	Form	Manufacturing country
A	*L. casei*<br>*L. rhamnosus*<br>*L. acidophilus*<br>*L. plantarum*<br>*L. helveticus*<br>*B. breve*<br>*B. longum*	5880<br>6600<br>4200<br>4200<br>120<br>4500<br>4500	Capsule	Canada
B	*L. acidophilus*<br>*L. plantarum*<br>*L. rhamnosus*<br>*L. gasseri*<br>*L. casei*<br>*B. animalis*	16000<br>5000<br>1000<br>1000<br>1000<br>6000	Capsule	Canada
C	*L. acidophilus*<br>*L. casei*<br>*L. rhamnosus*<br>*L. salivarius*<br>*B. bifidum*<br>*B. longum*	2700<br>300<br>300<br>300<br>2100<br>300	Tablet	Canada
D	*Lactobacillus*<br>*Bifidobacterium*<br>*Enterococcus*	10<br>10<br>10	Powder	China
E	*Lactobacillus*<br>*Bifidobacterium*<br>*Streptococcus*	5<br>50<br>5	Tablet	China
F	*L. reuteri*	20 / drop	Liquid	Canada

Note: *L.* = *Lactobacillus*; *B.* = *Bifidobacterium*
